# Brain basis for physical activity levels mediate beta Inhibition to improve cognitive function in elderly based on multimodality monitoring

**DOI:** 10.1038/s41598-025-98857-5

**Published:** 2025-05-23

**Authors:** Zixia Bu, Shan Jiang, Tingting Sun, Zhenxing Yang, Mo Sha, Fuqiang Dong

**Affiliations:** 1 School of International Chinese Language Education, Beijing Normal University, Beijing, China; 2 Department of Sports Science and Physical Education, The Chinese University of Hong Kong, Shatin, Hong Kong China; 3 Key Laboratory of Exercise and Physical Fitness, Ministry of Education, Beijing Sport University, Beijing, China; 4 College of Physical Education, Fuyang Normal University, Fuyang, China; 5 Sports Department, Communication University of China, Beijing, China; 6 College of Physical Education, Minzu University of China, Beijing, China

**Keywords:** Exercise, Cognitive function, Electroencephalogram, Event-related potential, Magnetic resonance imaging, Geriatrics, Lifestyle modification, Neurophysiology

## Abstract

The ameliorative effect of regular physical activity (PA) on cognitive function (CF) in older adults has been demonstrated. However, there are differences in the effects of different levels of PA on CF over time. To provide a more efficient exercise prescription for older adults, we compared differences in CF with neural mechanisms in individuals with different weekly activity levels. In this study, 68 older adults (*n* = 68, M = 47, F = 21, Age = 62.43 ± 4.36) were categorized according to the International Physical Activity Questionnaire (IPAQ) into high volume PA group (HG) (MET-min/week = 1516.64 ± 138.68), moderate volume PA group (MG) (MET-min/week = 1248.02 ± 119.86), and control group (MET-min/week = 1248.02 ± 119.86). 1516.64 ± 138.68), moderate volume PA group (MG) (MET-min/week = 1248.02 ± 119.86), and control group (MET-min/week = 846.78 ± 97.53). Differences in Stop Signal, Stroop, and 2-Back were compared among the three groups. Electroencephalogram (EEG), event-related potential (ERP), and magnetic resonance imaging (MRI) features were also observed. The results showed that for CF, 2-Back with Stroop was significantly higher in MG versus HG than in CG (F = 121.45, *P* < 0.01; F = 88.74, *P* < 0.01), and the opposite was true for response (F = 236.83, *P* < 0.01; F = 187.64, *P* < 0.01). Stroop was higher in HG than in MG (F = 38.78, *P* = 0.037) and Stop Signal was higher than in CG (F = 52.03, *P* < 0.01). In terms of EEG, the alpha band share of HG was higher than CG at rest (F = 41.34, *P* = 0.040) and the opposite in work (F = 25.67, *P* = 0.046). Although the remaining results were not statistically significant, PA levels were inversely proportional to resting midrange-band β (CG = 8.98 ± 1.02, MG = 8.07 ± 0.76, HG = 8.13 ± 0.88), and positively proportional to midrange and high-band β in WORK (midrange β: CG = 12.41 ± 2.29, MG = 12.98 ± 3.04, HG = 13.21 ± 2.85; high β: CG = 6.76 ± 2.23, MG = 7.35 ± 1.96, HG = 7.91 ± 2.3). In ERP, the latencies of N200, P300, and N450 were significantly shorter than those of CG in HG (F = 44.63, *P* = 0.041; F = 29.69, *P* = 0.045; F = 76.48, *P* < 0.01), and only N450 was shorter than that of CG in MG (F = 59.62, *P* < 0.01). On MRI, HG had Middle Frontal Gyrus (MFG) (HG > CG, voxel = 188, t = 3.72), Right Superior Frontal Gyrus (RSFG) (HG > CG, voxel = 238, t = 4.07), Left Cingulate (HG > CG, voxel = 343, t = 4.36), and Left Supplementary Motor Area (LSMA) (HG > CG, voxel = 343, t = 4.36) were activated more than CG. MFG (MG > CG, voxel = 172, t = 3.56), LSMA (MG > CG, voxel = 331, t = 4.28), and PHG (MG > CG, voxel = 204, t = 3.35) were activated more than CG. It was concluded that PA significantly enhanced CF in older adults, and that high volume PA was more effective than moderate volume. Specifically, VPA was found to be significantly more effective than MPA and LPA in enhancing individuals’ inhibition function, cognitive flexibility, working memory, and reaction speed. This may be attributed to the increase in ERP amplitude with shorter latency and greater gray matter density in frontal and temporal lobe regions.

**Supplementary Information:**

The online version contains supplementary material available at 10.1038/s41598-025-98857-5.

## Introduction

Cognitive function (CF) is defined as an individual’s ability to process information, memory, learning, and problem solving^1^. These abilities are crucial for an individual’s behavior in daily life and for adapting to the environment. Among the components of CF, working memory, response inhibition, and cognitive flexibility are particularly important^2^. These components determine the level of CF. However, CF decreases with age, and in severe cases, cognitive deficits may occur^3^. The proportion of the global elderly population over the age of 60, as defined by the World Health Organization (WHO), has reached 12% and is expected to reach 22% in 2050^4^. Consequently, the concern for cognitive health of the elderly has become a major research focus in the field of brain neuroscience.

A substantial body of evidence now indicates that physical activity (PA), as a non-pharmacological intervention, can be effective in slowing the decline of CF in older adults, leading to effects such as dementia prevention^5^. However, with the increasing signification of exercise, some studies have found that different levels of PA may differ in improving CF. For instance, WHO has classified PA into two categories: moderate physical activity (MPA) and vigorous physical activity (VPA). The WHO has emphasized that 150–300 min of MPA or 75–150 min of VPA per week not only improves EF in older adults, but also induces changes in brain plasticity^6^. Furthermore, Dong discovered that VPA was more efficacious in enhancing working memory than MPA^7^. Choi postulates that MPA may be more efficacious in reducing oxidative stress, glial activation, and cognitive impairment^8^. Conversely, Moritz observed that light physical activity (LPA) was more efficacious in enhancing cognitive flexibility and response inhibition, thereby facilitating the recovery of cognitive function in patients with Parkinson’s disease^9^. To date, although the beneficial effects of PA on CF have been well documented, the optimal dosage for slowing cognitive decline in older adults remains unclear.

In order to test the scientific dose of PA in the improvement of CF in older adults, Wang compared the differences in the effects of MPA and VPA on CF in older adults based on the Montreal Cognitive Assessment (MoCA) according to WHO criteria for the classification of PA^10^. However, CF involves many aspects, and working memory, response inhibition, and cognitive flexibility are the most important components. Consequently, in order to gain a more comprehensive and detailed understanding of the improvement effects of long-term regular PA on various aspects of CF and to reveal the neural structural mechanisms, the present study builds on Wang’s work by incorporating three groups of task paradigms into the experiment and utilizing multimodality monitoring (MMM) to reveal the neural mechanisms. The objective was to provide a more scientific and efficacious sports prescription for the improvement of CF in older adults. Based on the previous findings, the following hypotheses were proposed in the present study: (I) Individuals with long-term VPA may have higher cognitive paradigm scores than those with MPA. (II) Long-term VPA may result in significantly greater ERP amplitude and latency than MPA. (III) Individuals with long-term VPA may have greater gray matter volume than MPA.

## Materials and methods

### Participants

Sixty-eight participants were selected from the BABRI database. The inclusion criteria required participants to be over 50 years old and have a mini-mental status examination (MMSE) score greater than 24. All participants provided written informed consent. Exclusion criteria included neurological or psychiatric disorders, history of cardiovascular disease, muscular contraindications, and history of psychotropic drug addiction. Prior to the test, it was verified that the participants did not experience significant mood fluctuations or adverse physical conditions. The experiment received approval from the Human Experimentation Ethics Committee of the School of Physical Education and Sport at Beijing Normal University (authorization number: TY20220905, see Supplement 1 for further details). The experiment was conducted in accordance with the requirements and procedures of the Institutional Review Board. Demographic information is presented in Table 1.

**Table 1:** The characteristics of study population.

	Total	CG	MG	HG
Participants (n)	68	39	18	11
Male (n)	47	25	13	9
Female (n)	21	14	5	2
Age (years)	62.43 ± 4.36	61.79 ± 4.11	63.39 ± 4.80	63.09 ± 4.48
BMI (kg/m^2^)	22.2 ± 2.84	22.31 ± 2.79	22.04 ± 2.70	21.41 ± 3.31
Education (n)				
≥ 12 years	61	33	17	11
< 12 years	7	6	1	0
Smoking (n)	42	22	9	5
Alcohol (n)	55	29	12	8
Physical activity (MET-min/week)	1061.35 ± 288.37	846.78 ± 97.53	1248.02 ± 119.86	1516.64 ± 138.68
Sedentary behavior (h/week)	27.89 ± 10.08	29.43 ± 7.62	21.38 ± 5.61	21.63 ± 6.53

### Physical activity test

Physical activity was measured using the short version of International Physical Activity Questionnaire (IPAQ)^11^. IPAQ assigned metabolic equivalent (MET) corresponding to different intensities of PA by asking the study subjects about the frequency of the week (d/wk) and the time of day (min/d) for that intensity. The MET was 3.3 for walking, 4.0 for MPA, and 8.0 for VPA. The PA level for each intensity and the total PA level were calculated separately according to the formula.

The IPAQ has been comprehensively and maturely recognized as a suitable method for monitoring and assessing PA in older people. For instance, Claire utilized the IPAQ to evaluate the validity of moderate to vigorous PA and sedentary behavior in older adults in the UK, concluding that it exhibited high reliability and low error rates in Bland-Altman analyses. Subsequent to this, Teresa and T. Iona have applied the IPAQ to other European countries, also obtaining high Cronbach’s alpha results^12,13^. It has thus been demonstrated that IPAQ is a suitable tool for the assessment of PA levels in older adults.

Physical activity levels were categorized and grouped into 3 levels according to the criteria recommended by the IPAQ working group^10^: (1) High volume PA group (HG). VPA time ≥ 3d per week and total MET ≥ 1500, or PA time ≥ 7d per week and total MET ≥ 3000; (2) Moderate volume PA group (MG). Weekly VPA time ≥ 3d and daily PA time ≥ 20 min, or weekly MPA ≥ 5d and daily PA time ≥ 30 min, or weekly PA intervals ≥ 5d and total MET ≥ 600; (3) Control group (CG), with PA levels insufficient for the above criteria. The Cronbach α is 0.716 in the study.

The IPAQ formula is defined as follows:

① Walking MET × min/wk = 3.3 × Walking weekly frequency(d/wk) × Walking daily time(min/d).
② MPA MET × min/wk = 4.0 × MPA weekly frequency(d/wk) × MPA daily time(min/d).
③ VPA MET × min/wk = 8.0 × VPA weekly frequency(d/wk) × VPA daily time(min/d).
④ MVPA MET × min/wk = ② + ③.
⑤ Total PA MET × min/wk = ① + ② + ③.

## Measurements

### Executive function testing

**Fig. 1:**
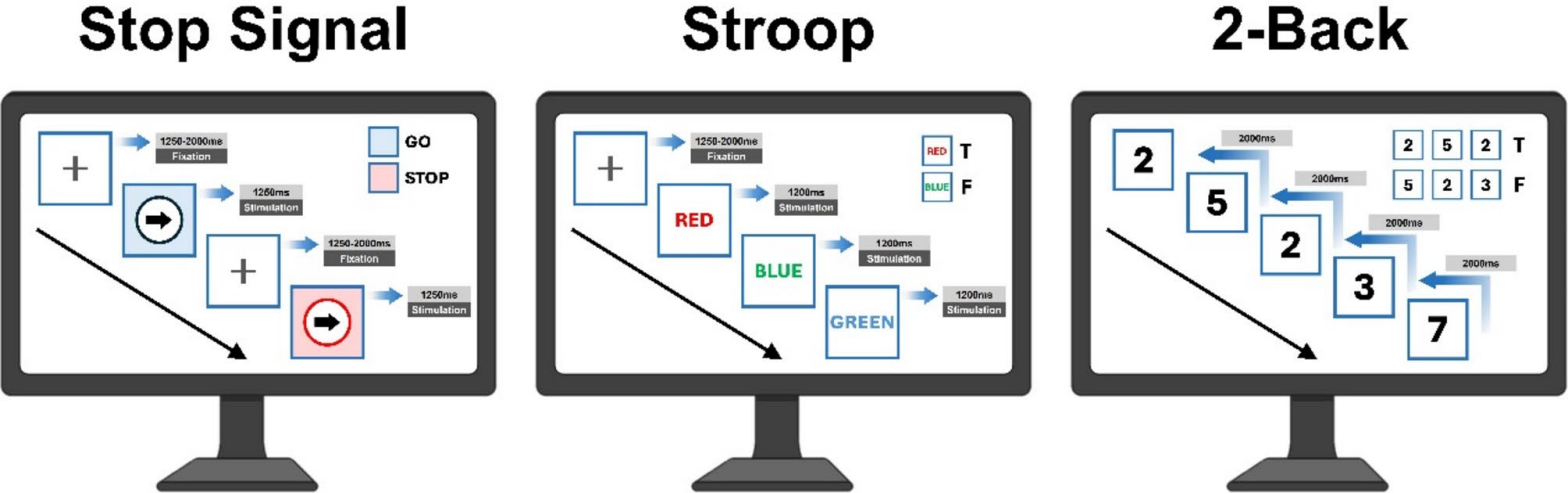
Operating procedure of the cognitive functional task paradigm. The Stop signal task is a tool employed in the assessment of cognitive functions, with a particular focus on the capacity for inhibition. This task requires participants to make correct or incorrect judgements based on the stimulus signal within a limited time frame. The Stroop task is utilised to evaluate cognitive flexibility, requiring participants to make correct or incorrect decisions based on the colour and the description of the stimulus signal given. The 2-Back task is utilised to assess working memory, with participants required to demonstrate consistent recall of the consistency of a stimulus signal that is updated at a constant rate. The amalgamation of these three tasks enables a comprehensive evaluation of an individual’s cognitive functioning.

The experiment consisted of three sessions, each testing a different type of task. There was a minimum of 72 h between each task. The CF was tested by the E-prime 2.0 software in three executions with three tasks: ① Stop signal task, ② Stroop task, ③ 2-Back task. The detail has been shown in Fig. 1.

In the Stop signal task, subjects were required to look at the ‘+’ at all times, and the duration of the signal ranged from 1250 milliseconds to 2000 milliseconds. During the GO stimulus, the colour of the circle and the arrow remained constant, and subjects were required to press the key corresponding to the direction of the arrow within 500 milliseconds. During the STOP stimulus, the circle turned red, and subjects were required to cease pressing the key. During STOP stimulation, the circle turned red and the subject had to stop pressing the key. The ratio of GO and STOP stimuli was 7:3, and the order of stimulation was randomized, each lasting 1200ms. The ratio of STOP stimuli was 7:3, and the order of stimulation was randomized, each lasting 1200ms.

In the course of the Stroop task, subjects were presented with three color stimuli: red, green, and blue. In the event that the color of the text corresponded to its meaning, the “T” key was to be pressed within a time frame of 100 milliseconds; conversely, if there was no correspondence, the “F” key was to be pressed. The ratio of correct to incorrect responses was 6:4, and the order of the stimuli was randomized.

In the 2-Back task, subjects were presented with a series of numbers in succession, with only a single number appearing in each stimulus, for a duration of 150ms, during which they were required to judge whether the current number was consistent with the number that preceded it. The “S” key was pressed for consistency, and the “L” key was pressed for inconsistency. The order of the digits was randomized.

### Electroencephalogram acquisition

Resting state EEG was recorded using a 32-channel brain wave analyzer (NeuroScan_32–128, Compumedics Neuroscan, Australia), with reference electrodes set to the default Oz during recording, and implementation filtering using a bandpass filter with an effective frequency interval of 0.01–100 Hz. The present study is specifically based on Wang’s study, which also evaluated signals from the frontal and central regions and applied the same equipment and parameter configuration^14^. Although Cz or mastoid electrodes were not used as the primary reference in this study, the actual Oz responses of the participants were the most significant in the filtered analyses and were more suitable for observing the stimulus sensations of the participants. Consequently, it was selected as the primary reference.

The sampling rate was 1000 Hz, and the scalp impedance of all electrodes was kept below 50k. 在In the course of the experiments, endeavors were made to minimize resistance through the replacement of conductive paste and the removal of hairs from the participants. Nevertheless, due to the substantial number of participants and the condition of the equipment, the actual resistance values exhibited significant variation. Consequently, this study incorporated at least two repeated measurements on the participants with the problem when available. Concurrently, the measured filter processing technique in MetLab software was utilized to minimize errors caused by resistance and artefacts, including EOG, power line interference, impedance fluctuation, and wire movement. Subjects were observed while performing tasks and while resting with their eyes closed, and their EEG was recorded. Furthermore, Brain Electrical Activity Mapping (BEAM) was recorded in this study to observe the potential characteristics of various brain regions in individuals with different levels of PA while performing the task.

The power spectrum of the EEG response was evaluated using the multi-step method, a method that reduces the variance of spectral estimates by multiplying the data with orthogonal cones known as Slepian functions. The power spectrum of the EEG response was evaluated using MATLAB _2022 Fourier transform was performed to extract specific spectra including α (10.0–13.0 Hz), midrange β (13.0–20.0 Hz), and high β (20.0–30.0 Hz), respectively.

### Event-related potential analysis

The neural electrical activity of subjects was observed in the present study after receiving stimuli during the task by event-related potential (ERP), and its neural mechanism was revealed. The ERPs involving the CF with three tasks, including N200, N450, P200, and P300, were considered based on previous studies. The frontal lobe loci were mainly used for observation, and the analysis indexes were amplitude and latency^15^.

The N200 component was filtered and recorded between 200-300ms, while the P300 was recorded between 300-500ms^16^. This ability typically manifests as the capacity to discern discrepancies or incongruities between stimuli, as well as the capability to filter out extraneous information. In the context of early target recognition, it has been demonstrated to facilitate the rapid detection of mismatches or target stimuli. The P200 component was recorded within the 250-350ms time frame. It has been suggested that this primarily reflects rapid attentional allocation to sensory input, particularly the initial processing of salient features. The initial emotional processing of faces and emotional information may be associated with rapid sensitivity to such information. The P300 is a cognitive neurophysiological response that occurs within 200-400ms of the stimulus. It has been demonstrated that the P300 reflects three main aspects: the updating of working memory content, the allocation of the individual’s attentional resources to the target stimulus and the assessment of the relevance of the stimulus to the current task. The N450 time window, which is typically associated with semantic conflict processing, typically reflects an individual’s efforts to resolve conflict in a given task. It has been associated with higher levels of executive functioning, such as the regulation and control of conflict responses, during executive functioning and cognitive control^17^.

These metrics were observed in the central frontal lobe sites (Fz, FCz, F3, F4, FC1, and FC2). The study compared the waveform differences of each potential in the time domain based on population differences and selected the most prominent visual features for comparison. The study observed that the frontal ERP component occurred centrally at Fz, which is consistent with previous findings^18^.

### MRI acquisition and processing

A human MRI scanner (Trio 3.0T, Siemens, Germany) was used for image acquisition in this study. Concurrently, the present study comprehensively referenced Li’s experiments and adjusted the device parameters to align with the parameters employed in his study^19^. T1 structural images were acquired using a fast gradient echo sequence with a repetition time of 1900ms, an echo time of 3.44ms, a flip angle of 9°, a field of view of 256 mm×256 mm, a number of slices of 176, and a thickness of 1 mm.

The present study utilized high-resolution T1-weighted imaging data, which was subjected to pre-processing using the CAT12 (Computational Anatomy Toolbox) toolbox of the SPM12 software package with a view to the removal of motion and other artefacts. CAT12 is widely regarded as a more accurate tool than VBM8 for the analysis of volume changes^20^. The images of all participants were segmented into white matter, grey matter and cerebrospinal fluid. Thereafter, the images were normalized through the standard space of the Montreal Neurological Institute (MNI), and parameters were adjusted using the Diffeomorphic Anatomical Registration Through Exponential Lie Algebra (DARTEL) toolbox for parameter tuning^21^. Following a thorough evaluation of the quality using CAT12 to rectify any discrepancies, the residual grey matter images were processed using a full width at half maximum (FWHM) Gaussian kernel to ensure smoothness.

Meanwhile, the MRI data were pre-processed using the VBM software, and the T1 structural image consisted of data transformation, spatial normalization (non-linear alignment of images using DAR-TEL followed by alignment to the gray matter template in MNI space), image segmentation (white matter, gray matter, cerebrospinal fluid), and spatial smoothing (Gaussian smoothing with a full width at half maximum of 8 mm). The methodology employed in this study involved the use of 8 mm thick slices to facilitate clear comparison of the different areas included in the study.

### Voxel-based morphological analysis

Structural MRI-based gray matter morphology analysis employs high-resolution T1-weighted imaging. Voxel-based analysis (VBM) is an automated morphometric analysis method for whole-brain voxel-by-voxel comparisons of T1-weighted structural images, enabling the identification of differences in the gray matter volume of localized brain regions. VBM has been extensively utilized in the analysis of neuroanatomical images due to its numerous advantages^22^. VBM analysis is straightforward to manipulate and can identify intergroup differences in the microstructure of the living brain for whole-brain analysis. Prior to statistical analysis, structural image data must undergo preprocessing. Firstly, the structural image is segmented into gray matter, white matter, and cerebrospinal fluid. Secondly, the image is normalized to the same standard template to correct for differences in individual head shapes. Secondly, the gray matter volume is corrected to obtain the absolute amount of gray matter. Finally, the image is spatially smoothed to ensure normal distribution of the data and to reduce inter-individual variability. The smoothed data were statistically analyzed at the voxel level.

### Statistical analysis

The study was statistically and analytically performed using IBM SPSS Statistics_26, normality test followed by descriptive statistics and reliability test. All data were presented as mean ± standard deviation. One-way analysis of variance (ANOVA) was utilized to compare and evaluate the behavioral and absolute voltage amplitude data of participants in the reference group. Gray matter images were then subjected to a two-sample t-test using SPM12, and the statistics were corrected for false discovery rate (FDR). The Pearson correlation coefficient was utilized to analyse the correlation between individuals’ demographic characteristics, behavioral data and voxel-based morphological data. The significance level for correlation analysis was set at 0.05, and FDR method was employed for correction. Differential gray matter density values of brain regions were correlated with each cognitive function index. Data following normal distribution were analyzed by linear Pearson correlation coefficient, and data not following normal distribution were analyzed by nonlinear Spearman’s rank correlation coefficient, with the significance threshold set at *P* < 0.05.

## Results

### Dose differences and EEG characteristics of physical activity in improving executive function

The results showed that the three groups of subjects differed in task performance but had similar EEG components. ANOVA post-hoc analyses revealed that PA mediated higher task scores with shorter response times in older adults, and that the level of PA was positively related to this effect. In 2-Back, CG was significantly less correct than MG vs. HG (F = 121.45, *P* < 0.01; F = 88.74, *P* < 0.01), and the opposite was true for reaction time (F = 236.83, *P* < 0.01; F = 187.64, *P* < 0.01), but there was no significant between-group difference in PA between the two groups. In Stop signal, only HG was more correct than CG (F = 52.03, *P* < 0.01), but all three were similar when responding. In Stroop, not only MG and HG showed higher accuracy and shorter response time than CG (F = 97.56, *P* < 0.01; F = 134.68, *P* < 0.01; F = 179.43, *P* < 0.01; F = 185.39, *P* < 0.01), but also HG had a higher correctness rate than MG (F = 38.78, *P* = 0.037).

**Fig. 2:**
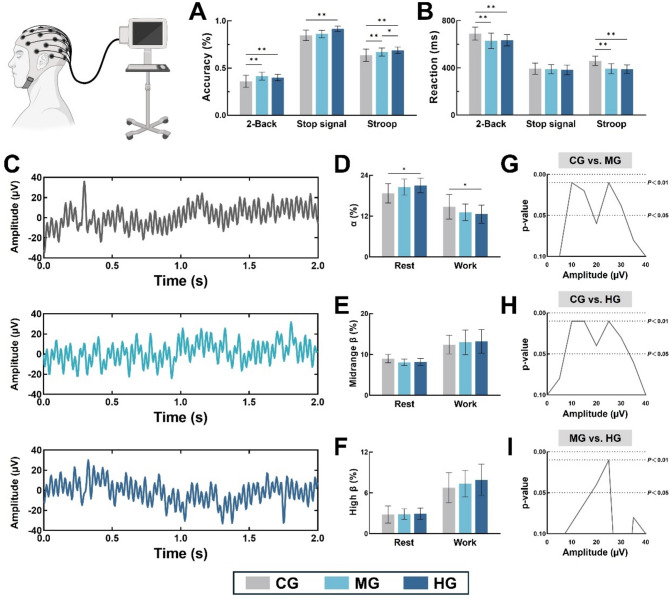
Characterization of cognitive function and neuroelectrical activity in elderly with different PA levels. (**A**, **B**) Task accuracy and response time in elderly with different levels of PA; (**C**) EEG monitoring in elderly with different PA levels; (**D**–**F**) Component proportion of α, midrange and high β at rest and task; (**G**–**I**) Amplitude comparisons across the full band for the three groups in the task. As demonstrated in this figure, subjects designated MG and HG exhibited elevated 2-Back scores and reduced reaction times in comparison to the CG (*P* < 0.01). Furthermore, HG demonstrated a higher score for Stop Signal in comparison to the other two groups (*P* < 0.01). In addition, a comparative analysis revealed that MG and HG exhibited higher Stroop scores and faster reaction times in comparison to CG (*P* < 0.01). Additionally, HG demonstrated a higher score than MG (*P* = 0.037).

The results of the EEG components in each band showed that the proportion of alpha waves was significantly higher in HG than in CG in rest (F = 41.34, *P* = 0.040), and the opposite in WORK (F = 25.67, *P* = 0.046). Although midrange and high β were not statistically significant across the three groups, it was evident from the readings that elevated levels of PA mediated lower midrange-band β in the older adults in the REST state (CG = 8.98 ± 1.02, MG = 8.07 ± 0.76, HG = 8.13 ± 0.88) in contrast to a greater degree of in-task high-band β oscillations (CG = 6.76 ± 2.23, MG = 7.35 ± 1.96, HG = 7.91 ± 2.3). The results of amplitude differences in each band showed that the differences in MG were mainly in the mid-frequency (10–15 Hz) and high-frequency (25–30 Hz) bands compared with CG, while the differences in HG were reflected in the full band (10–30 Hz).The results of the PA intergroup control showed that the differences in MG and HG were mainly in the range of 20–25 Hz, with the rest of the bands having similar levels of expression. For details, see Table 2; Fig. 2.

**Table 2:** Effects of regular PA on cognitive function and neuroelectrical responses in elderly.

	CG	MG	HG
2-Back			
Accuracy (%)	0.3597 ± 0.0647	0.4133 ± 0.0406	0.3989 ± 0.0342
Reaction (ms)	689.73 ± 55.68	628.69 ± 64.49	633.46 ± 48.37
Stop signal			
Accuracy (%)	0.8474 ± 0.0545	0.8612 ± 0.0396	0.9154 ± 0.0271
Reaction (ms)	392.67 ± 46.32	388.73 ± 37.59	383.28 ± 41.4
Stroop			
Accuracy (%)	0.6356 ± 0.0658	0.669 ± 0.0438	0.6876 ± 0.0332
Reaction (ms)	458.91 ± 39.45	391.56 ± 43.28	388.65 ± 36.43
α			
Rest (%)	18.68 ± 2.82	20.51 ± 2.37	21.01 ± 2.14
Work (%)	14.74 ± 3.59	13.12 ± 2.44	12.58 ± 2.67
Midrange β (%)			
Rest (%)	8.98 ± 1.02	8.07 ± 0.76	8.13 ± 0.88
Work (%)	12.41 ± 2.29	12.98 ± 3.04	13.21 ± 2.85
High β (%)			
Rest (%)	2.82 ± 1.24	2.87 ± 0.78	2.93 ± 0.84
Work (%)	6.76 ± 2.23	7.35 ± 1.96	7.91 ± 2.3
N200			
Amplitude (µV)	− 1.65 ± 0.71	− 1.89 ± 0.68	− 1.91 ± 0.77
Latency (ms)	245.62 ± 18.79	225.48 ± 15.53	218.34 ± 17.45
N450			
Amplitude (µV)	− 3.12 ± 0.82	− 3.35 ± 0.65	− 3.29 ± 0.77
Latency (ms)	493.64 ± 35.2	459.32 ± 28.94	448.57 ± 28.78
P200			
Amplitude (µV)	3.73 ± 0.73	3.79 ± 0.81	3.85 ± 0.69
Latency (ms)	198.38 ± 18.93	193.65 ± 17.84	190.46 ± 20.12
P300			
Amplitude (µV)	4.56 ± 0.62	4.7 ± 0.69	4.67 ± 0.84
Latency (ms)	332.16 ± 28.69	315.41 ± 22.34	308.27 ± 20.65

### ERP differences mediated by level of physical activity

**Fig. 3:**
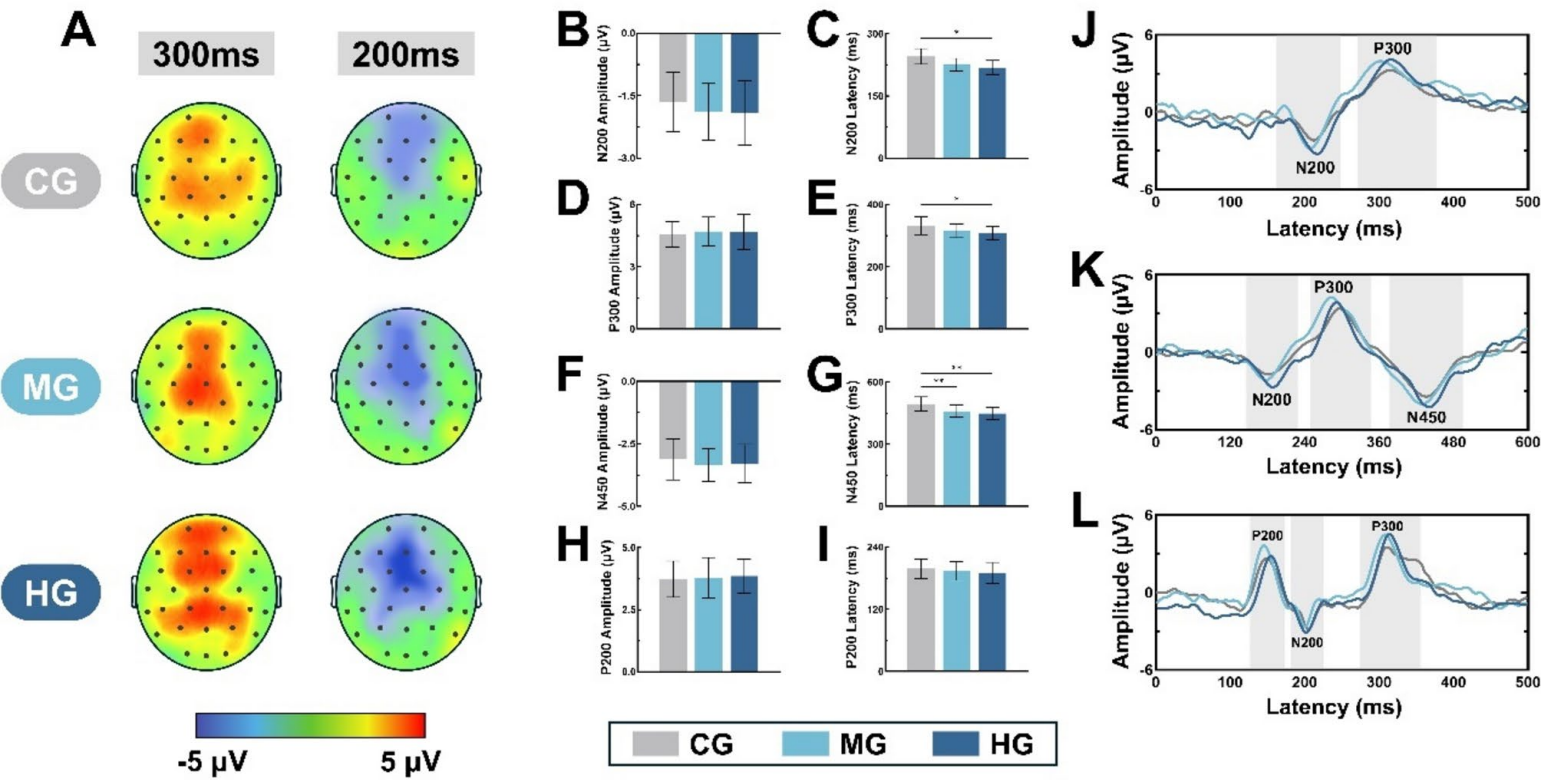
ERP and BEAM characteristics in task performance in elderly with different PA levels. (**A**) BEAM in the task for three groups of participants; (**B**, **C**) Amplitude and latency of N200 in three groups; (**D**, **E**) Amplitude and latency of P300 in three groups; (**F**, **G**) Amplitude and latency of N450 in three groups; (**H**, **I**) Amplitude and latency of P200 in three groups; (**J**–**L**) Time-domain characterization of potentials in three groups with Stop signal, Stroop, and 2-Back. As demonstrated in this figure, the latencies of N200, P300, and N450 were found to be significantly reduced (*P* < 0.05) for HG compared to CG versus MG. Furthermore, the latency of N450 was found to be significantly lower in MG compared to CG (*P* < 0.01).

The results showed that there was little difference in ERP amplitudes among the three groups of participants, but there were differences in the latencies of some of the potential. ANOVA post-hoc analyses showed that although there were no statistical differences in N200, P300, N450, and P200 amplitudes among the three groups of individuals, it was evident from the readings that MG and HG had higher amplitudes of each potential than CG in the performance of the task. Among them, in N200 and P200 the higher the PA level, the greater the difference in amplitude (N200: CG=-1.65 ± 0.71, MG=-1.89 ± 0.68, HG=-1.91 ± 0.77; P200: CG = 3.73 ± 0.73, MG = 3.79 ± 0.81, HG = 3.85 ± 0.69). However, in P300 and N450, MG amplitude was higher than HG (P300: CG = 4.56 ± 0.62, MG = 4.7 ± 0.69, HG = 4.67 ± 0.84; N450: CG=-3.12 ± 0.82, MG=-3.35 ± 0.65, HG=-3.29 ± 0.77). The latency results showed that the N200, P300, and N450 latencies were significantly shorter for HG than for CG (F = 44.63, *P* = 0.041; F = 29.69, *P* = 0.045; F = 76.48, *P* < 0.01), whereas the MG was significantly shorter than CG only in N450 (F = 59.62, *P* < 0.01). As can be seen by BEAM, long-term PA increased the amplitude level in older adults during the task with changes in voltage volts in the central, parietal, and frontal regions. See Table 2; Fig. 3 for details.

### Physical activity improves the plasticity of brain structures

MRI results showed that long-term regular PA significantly elevated gray matter volume in the frontal and parietal brain regions of the elderly. Among them, MFG, LSMA, and PHG of MG and HG were greater than that of CG, and the most significant difference was found in LSMA. However, for RSFG, LCG, LACG, and RSMG, only HG was greater than CG, and the difference was most significant for RDFG. Meanwhile, there was no significant difference in gray matter volume between MG and HG. The voxel-based morphological result showed that in the PA group, the area of the red activated region was larger than that of CG in the − 40 mm to -10 mm cross section versus the 35 mm to 55 mm cross section. This suggests that the gray matter density of MG and HG is higher than that of CG.

**Fig. 4:**
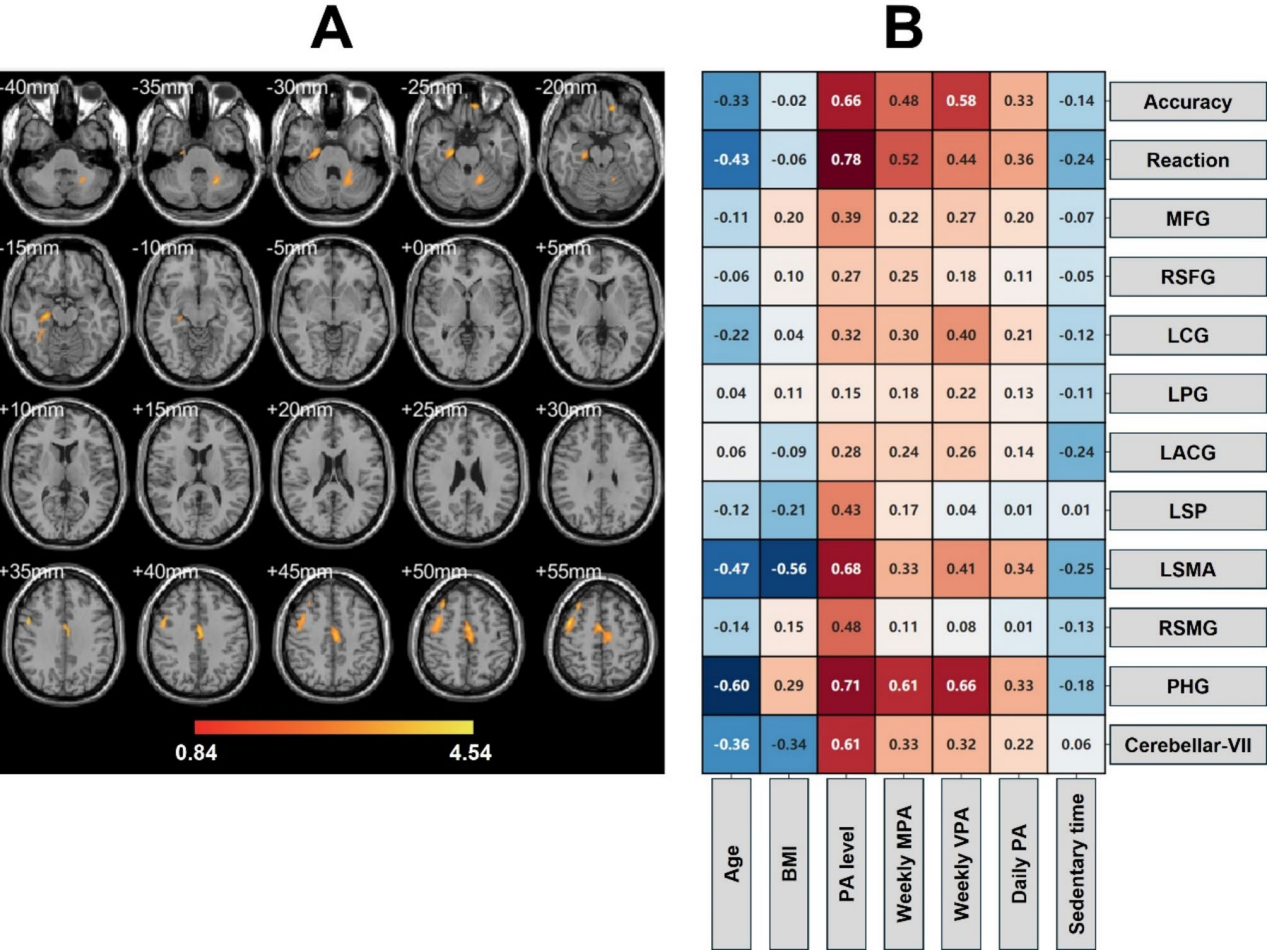
Effect and correlation of PA on gray matter volume of the brain in elderly. (**A**) The effect of regular PA on the volume of elderly gray matter; (**B**) Correlation between PA levels and gray matter volume.

This phenomenon also suggests that the improvement effect of long-term regular PA on cognitive function in older adults may be related to the enhancement of the connectivity of static brain networks. Correlation results showed that the total PA level, weekly MPA and weekly VPA measures were significantly positively correlated with PHG, LSMA, accuracy, and reaction time, and the effect size of VPA was higher than that of MPA, which suggests that the higher the level of PA, the greater the gray matter density in older adults, and that VPA was more effective than MPA. In addition, accuracy, reaction time, LSMA, and PHG were significantly negatively correlated with age and BMI, and were most affected by age. This suggests that an increase in AGE and BMI may affect gray matter density in older adults. See Table 3; Fig. 4 for details.

**Table 3:** Effects of PA on Gray matter volume in elderly.

Position	Comparison	BA	Hemisphere	MNI coordinate (mm)	Cluster size (voxel)	t
MFG	MG> CG	BA 6	Left	53, − 15, 6	172	3.56
	HG> CG				188	3.72
	HG> MG				NS	
RSFG	MG> CG	BA 4, 8	Right	29, 8, 62	NS	
	HG> CG				238	4.07
	HG> MG				NS	
LCG	MG> CG	BA 24	Left	− 8, − 35, 36	NS	
	HG> CG				145	3.25
	HG> MG				NS	
LPG	MG> CG	BA 6	Left	− 26, − 30, 72	NS	
	HG> CG				NS	
	HG> MG				NS	
LACG	MG> CG	BA 6	Left	− 51, 6, 32	NS	
	HG> CG				133	3.06
	HG> MG				NS	
LSP	MG> CG	BA 40	Left	− 35, − 54, 66	NS	
	HG> CG				NS	
	HG> MG				NS	
LSMA	MG> CG	BA 6	Left	− 6, − 15, 53	331	4.28
	HG> CG				343	4.36
	HG> MG				NS	
RSMG	MG> CG	BA 40	Left	65, − 45, 38	NS	
	HG> CG				114	3.14
	HG> MG				NS	
PHG	MG> CG	BA 35	Right	27, − 26, − 23	204	3.35
	HG> CG				258	3.48
	HG> MG				NS	
Cerebellar-VII	MG> CG		Right	− 20, − 66, − 42	NS	
	HG> CG				NS	
	HG> MG				NS	

*BA* Brodmann area, *MFG* middle frontal gyrus, *RSFG* right superior frontal gyrus, *LCG* left cingulate gyrus, *LPG* left postcentral gyrus, *LACG* left anterior central gyrus, *LSP* left superior parietal, *LSMA* left supplementary motor area, *RSMG* right supramarginal gyrus, *PHG* parahippocampal gyrus.

## Discussion

The efficacy of long-term regular PA in improving CF in older adults has been extensively documented, yet the optimal dosage profile and underlying structural basis in various aspects of CF remain uncertain. While some studies have indicated that the effects of VPA may be superior to other forms of exercise, there is considerable heterogeneity between studies, and the neural mechanisms remain poorly understood. This study builds upon previous research to examine the differential effects of VPA and MPA on cognitive function in older adults, with a focus on the physiological characteristics of intracranial gray matter structures across diverse populations. The objective is to develop a more rational and effective exercise prescription for this age group, with a rationale grounded in physiological mechanisms.

The initial findings of this study indicated that regular PA significantly enhanced CF in older adults. However, the effect of VPA on response inhibition and cognitive flexibility was more pronounced. In this study, the 2-Back and Stroop scores of HG and MG were higher than those of CG, and the response time was shorter than that of the CG. However, the stop-signal and Stroop scores of the HG were higher than those of the MG. This suggests that VPA enhances the efficiency of CF improvement in older adults. Consequently, hypothesis (I) can be accepted, which is consistent with previous results. Anabela conducted a longitudinal study to examine the effects of a short period of time (one week) of moderate-to-vigorous physical activity (MVPA) on executive function in older adults. The results demonstrated that the PA group outperformed the sedentary control group in processing speed and language function after the intervention^23^. With regard to memory, Wang observed that 12 weeks of MPA (Tai Chi) significantly enhanced older adults’ 2-back task accuracy and reaction time, thereby enhancing their working memory^24^. To further verify whether PA level leads to differences in CF in older adults, Li analyzed the correlation between PA level and CF. The results indicated a positive correlation between muscle strength, PA level, and memory and cognitive flexibility in older adults^25^. This also implies that elevating PA levels is an effective way to improve CF. To substantiate this assertion, Wang conducted a cross-sectional survey of 647 older adults aged 60 and above, categorizing them according to their levels of PA in terms of visuospatial, attention, language, abstraction, and delayed recall. The results demonstrated a positive correlation between PA and CF scores, with a saturation effect between the two variables^10^. In contrast, Paula, after a comprehensive evaluation of 104 studies, found that while PA may mitigate the decline in CF in older adults, all follow-ups of more than 10 years showed no significant association^26^. In conjunction with the present results, this study hypothesizes that age may have contributed to the gradual attenuation of the long-term regular PA effect. Paula included a large number of studies in which participants were older than 76, a range that exceeds the WHO limit of Very Old^27^. In contrast, the sample in this study was between 60 and 65 years old, which may have resulted in an underestimation of the effect of PA on CF. Collectively, these findings suggest that age is not only a crucial factor in determining the effectiveness of PA, but also increases the associated risk of exercise. In conclusion, while increasing PA levels can be an effective means of maintaining CF in the retired population, the key factor is to maintain a long-term PA habit in the early years to build a foundation of physical fitness for exercise in old age.

The second finding of the present study was that VPA demonstrated superior efficacy in improving CF, which may be related to higher alpha occupancy in the resting state and suppression of mid-frequency beta with higher high-frequency β-oscillation in the task. Although VPA resulted in greater frontal-to-central ERP amplitude, there was no significant difference in terms of shorter latency compared with MPA. In the present study, exercise intensity was found to be positively associated with resting state alpha occupancy and mid- and high-frequency β-oscillation in the task, and inversely associated with resting state mid-frequency β-band activity and α-oscillation in the task. In contrast, PA resulted in positive and negative potential amplitude in a larger area of frontal, parietal, and central regions in the older adults’ task, significantly shortening the latencies of P300, N200, and N450. Although VPA-mediated individuals exhibited a greater maximal ERP amplitude, the latency was not significantly shorter than that of MPA. Therefore, hypothesis (II) could not be fully accepted. This is consistent with some of the findings of Wang, who found that, compared with MPA, long-term VPA not only significantly elevated individuals’ executive function and suppressed midrange β-band activity during quiet time, but also improved the body’s central tolerance to fatigue and stress stimuli^14^. Similarly, Numan compared the EEG characteristics of professional dancers and fast-ball sports athletes and found that the fast-ball athlete exhibited a greater alpha band amplitude at rest and a higher level of β-oscillation than the dancer during the task^28^. Zhang demonstrated that the mediating effect of high β-oscillation in cognitive processes was significant only after prolonged VPA, which may be an important mechanism by which VPA reverses the decline in CF brought about by aging^29^. In terms of ERP, Patelaki’s findings indicated that long-term PA resulted in individuals exhibiting greater positive amplitude of the P200 and P300 in the frontal and central regions during a memory task. This attenuation pattern was hypothesized to reflect a more diligent recalibration of neural processes following long-term PA^30^. Furthermore, Chang demonstrated that a single session of PA can have a positive effect on CF. He discovered that individuals exhibited significant improvements in their Stroop scores and reaction times following acute exercise. Moreover, he observed that the N100, N200, and P300 components of the process exhibited the most pronounced changes^31^. It is noteworthy that Ludyga demonstrated a correlation between negative values of N200 and N450 and participation in open-skill sports. This was evidenced by the observation that individuals who engaged in such PA for an extended period of time exhibited higher Stroop and Stop Signal scores than those who engaged in closed-skill sports^17^. This effect was also observed in the present study with regard to VPA. Therefore, it can be postulated that incorporating open-skill sports into weekly PA may further enhance the improvement of CF. In contrast to the above evidence, the present study used PA level as a variable for the first time and found that older adults with long-term VPA had higher N200 and P200 amplitude fluctuations than MPA and had shorter latencies. This also shows that VPA improves working memory and cognitive flexibility better than MPA, and the neural mechanism may be related to N200 and P200 amplitude variability with shorter latency.

The third finding of the present study was that regular PA improved gray matter density in older adults, whereas VPA had a more significant stimulatory effect on frontal and temporal lobe regions. In the present study, long-term MPA and VPA resulted in greater amplitude of MFG, RSFG, LCG, LACG, LSMA, RSMG, and PHG densities in older adults. The effects of VPA on RSFG, LCG, LACG, and RSMG areas were greater than those of MPA, in which RSFG and LCG are generally associated with higher cognitive functions and are involved in behaviors such as control of external stimuli, attentional control, and pain perception^32^, whereas LACG and RSMG play important roles in spatial perception, hand coordination, and language comprehension, and also play an important role in fine motor control^33^. This also implies that older adults with long-term VPA will have higher levels of executive functioning. Therefore, hypothesis (III) can be accepted. The above phenomenon can be explained in terms of neural mechanisms. Chen’s systematic evaluation demonstrated that at least three months of VPA can optimize the activity status of CF-related brain regions (anterior cingulate gyrus, middle anterior gyrus, and supraparietal lobule, etc.) and increase prefrontal cortical volume in older adults^34^. To elucidate the mechanisms underlying the beneficial effects of PA on memory function, Steventon observed that, following a single week of PA in older adults, individuals exhibited elevated voxel counts in the corpus callosum, uncinate fasciculus, and parahippocampal cingulum relative to baseline levels, accompanied by selective increases in hippocampal blood flow. This evidence suggests that brief periods of PA can induce alterations in intracranial microstructure and vascular brain^35^. Similar to the present study, Liu, after comparing the effects of two mind-body exercises (Tai Chi and Baduanjin) on various gray matter structures in older adults from the perspective of resting-state functional connectivity of brain networks, found that the two types of PA significantly increased the resting-state functional connectivity (rsFC) of the posterior cingulate cortex with the right putamen/caudate, but individuals with longer habituation showed greater rsFC between the medial prefrontal cortex (mPFC) and the right putamen/caudate volume. PA levels and rsFC with the orbital prefrontal gyrus were negatively correlated with the Visual Reproduction subscore^36^. Despite the beneficial effect of PA on gray matter density in older adults, exercise-induced fatigue due to transitioning from one type of PA to another can have a negative impact. Koevoets used the timeliness of PA as a variable and found that six months of PA did not result in an increase in hippocampal subfield and cortical thickness or gray matter volume in older adults, despite improving CF. However, the prevalence of high fatigue in their included sample is a cause for concern^37^. In the context of the present study, we conducted the first comparison of gray matter density differences between older adults under long-term MPA and VPA by MRI. Our findings indicated that although long-term VPA resulted in greater structural improvement of gray matter in frontal and temporal lobe regions, controlling the amount of exercise is a key factor in exerting this effect. The present study thus indicates that the course of VPA necessitates the implementation of a training program that is continuously adapted to the individual’s subjective level of fatigue, thereby preventing the occurrence of exercise fatigue and exercise-related injury.

In conclusion, increasing weekly PA levels can enhance CF to a greater extent in older adults, and its neural mechanisms are more complex. With regard to endogenous mechanisms, the VPA group demonstrated greater α-band activity during quiet than controls, which was more significant than the high β-oscillation of MPA. On a structural basis, PA significantly enhanced gray matter density in older adults, but greater gray matter volume in frontal and temporal regions was observed in the VPA population. Consequently, VPA was superior to MPA in the promotion of CF. However, given that the focus is on older adults, it is important to consider the physiological limitations of this group. Excessive or inappropriate training programs may increase the probability of injury and fatigue. Therefore, based on Principle of Appropriate Load^38^ and Principle of Motivation in Sports^39^, it is recommended that older adults use the rating of perceived exertion (RPE) to quantify their own exercise sensation, with the aim of improving as much as possible within the limited scope of the PA level. Furthermore, the Feeling Scale (FS) can be employed to assess the psychological state at different points in time, thereby enabling the program to be adapted in a timely manner in order to optimize exercise motivation.

### Limitations and suggestions for future research

The present study aimed to investigate the effects of long-term PA on CF in older adults from the perspectives of working memory, response inhibition, and cognitive flexibility. Additionally, the study sought to elucidate the underlying neural mechanisms and structural bases of these effects through ERP and MRI techniques. Furthermore, the differences in the effects of MPA and VPA on CF were also compared. The objective was to identify more efficient exercise prescriptions to enhance exercise efficiency in older adults. Although this study employed a cross-sectional design to compare the characteristics of the three populations in terms of CF, EEG, ERP, and MRI, through a cohort study, and evaluated them according to the results, several limitations remain.

Firstly, it is necessary to define the age range of the participants. In this study, 68 older adults were included as subjects, all of whom were between the ages of 60 and 65. This age range is qualified by the WHO as the stage of initial aging. Older adults in this age group are in better physical condition relative to those over 70 years of age, thus supporting the implementation of different levels of PA. However, the probability of risk for PA increases with age. Consequently, if the study is focused on older adults, it is important to consider the reality of implementing PA in the 70 + age group. Future research should therefore address the uncertainty of PA with age.

Secondly, the issue of analyzing methods of MRI must be addressed. In general, conventional MRI and fMRI studies should establish a prediction model for the effect of different elements on gray matter volume by singular value decomposition (SVD), latent vector (LV), and structural covariance network analysis based on the number of voxels of different variables with MRI. However, this method has certain characteristics, such as only being able to compare the differences between two groups of variables. However, the present study included three groups of variables, and the difference between the effects of MPA and VPA on gray matter density was minimal, allowing for clear observation of the difference in volume from the MRI images. This phenomenon can also be seen in Table 3 of this study. Consequently, in this study, the MRI results section did not include images of the difference in gray matter structure between MPA and VPA. Instead, these differences were enumerated in Table 3.

In future studies, we will therefore focus primarily on heterogeneous variations for older adults across the age range. Furthermore, we will divide the PA forms based on intensity, program, or open/closed characteristics, such as HIIT vs. MICT or basketball or running. Concurrently, the fMRI method will be incorporated into the study, which will permit a more intuitive comprehension of the activity and volume characteristics of individual brain regions under different conditions, and will provide more accurate and authoritative exercise prescriptions for the cognitive health of the elderly.

## Conclusion

Long-term regular PA significantly improved CF in older adults, whereas VPA was superior to MPA. Increasing the intensity and duration of weekly PA benefited CF in older adults. In particular, VPA significantly outperformed MPA and LPA in improving inhibitory function, cognitive flexibility, working memory and reaction speed. Therefore, it is recommended that older adults try to increase their PA levels as much as possible, within their individual tolerance range, to achieve the goal of effective exercise. The reasons for this may be attributed to the increase in ERP amplitude with shorter latency and greater gray matter density in frontal and temporal lobe regions. However, it is crucial to emphasize that reasonable control of the amount of exercise is the key factor. It is therefore recommended that older adults pay real-time attention to their exercise perception and fatigue level.

## Electronic supplementary material

Below is the link to the electronic supplementary material.

Supplementary Material 1

Supplementary Material 2

## Data Availability

Data are available upon reasonable request. The datasets used and/or analyzed during the current study are available from the corresponding author upon reasonable request.
